# Crc Regulates Succinate-Mediated Repression of Mineral Phosphate Solubilization in *Acinetobacter* sp. SK2 by Modulating Membrane Glucose Dehydrogenase

**DOI:** 10.3389/fmicb.2021.641119

**Published:** 2021-07-12

**Authors:** Krishna Bharwad, Niharika Ghoghari, Shalini Rajkumar

**Affiliations:** Institute of Science, Nirma University, Ahmedabad, India

**Keywords:** *Acinetobacter* sp. SK2, succinate, MPS, sGDH, mGDH, Crc

## Abstract

The plant growth-promoting *Acinetobacter* sp. SK2 isolated from *Vigna radiata* rhizosphere was characterized for mineral phosphate solubilization (MPS). To understand the contribution of the membrane glucose dehydrogenase (mGDH) and soluble glucose dehydrogenase (sGDH) in glucose oxidation and MPS, insertional inactivation of the corresponding genes was carried out. The disruption of mGDH encoding gene *gdhA* resulted in complete loss of mGDH activity, which confirmed its role in periplasmic glucose oxidation and gluconate-mediated MPS phenotype. The inactivation of sGDH encoding gene *gdhB* resulted in loss of sGDH activity, which did not alter the MPS or mGDH activity. Thus, it was also concluded that the sGDH was dispensable in gluconate-mediated MPS. Supplementation of succinate in glucose-containing medium suppressed the activity of mGDH (and sGDH) and therefore repressed the MPS phenotype. The catabolite repression control protein (Crc) of *Pseudomonas* was implicated in *Acinetobacter* sp. for a similar function in the presence of preferred and non-preferred carbon sources. To understand the regulatory linkage between Crc and genes for glucose oxidation, *crc* mutants were generated. The inactivation of *crc* resulted in increased activity of the mGDH in glucose + succinate-grown cells, indicating derepression. An increase in phosphate solubilization up to 44% in glucose + succinate-grown *crc*^–^ compared with glucose-grown cells was recorded, which was significantly repressed in the wild-type strain under similar conditions. It is therefore proposed that in *Acinetobacter* sp. SK2, Crc is involved in the succinate-provoked repression of the MPS phenotype. The gene expression data indicated that Hfq may also have a regulating role in preferential utilization of carbon source by perhaps modulating Crc–Hfq functionality. *V. radiata* plants inoculated with the wild type improved both root and shoot length by 1.3 to 1.4-fold. However, *crc*^–^ increased the root and shoot length by 1.6-fold, compared with the uninoculated controls. In mimicking the soil condition (in the presence of multiple carbon sources, e.g., succinate along with glucose), the *crc*^–^ strain of *Acinetobacter* sp. SK2 performed better in supporting the growth of *V. radiata* in pot experiments.

## Introduction

The metabolically versatile genus *Acinetobacter* can thrive in diverse environments including rhizosphere and can be exploited to support plant growth (Young et al., 2005). *Acinetobacter* sp. SK2 isolated from mung bean rhizosphere released soluble P from complex sources. Besides being a phosphate solubilizer, it was also reported for other plant growth promotion (PGP) activities like indole-3-acetic acid (IAA), siderophore, and hydrogen cyanide (HCN) production (Bharwad and Rajkumar, 2020). In a previous study, we have reported that the mineral phosphate solubilization (MPS) ability of the isolate is attributed to gluconate secretion when grown on glucose. However, the acid production and hence the MPS phenotype were repressed by succinate when present alone or in combination with glucose. The gluconate production was facilitated by membrane glucose dehydrogenase (mGDH) and possibly had a contribution from soluble glucose dehydrogenase (sGDH). The activity of enzymes (mGDH and sGDH) and the expression of the corresponding genes were significantly lower in the presence of succinate or succinate along with glucose. The preference of succinate over glucose was effected by repression of glucose oxidation genes in the isolate *Acinetobacter* sp. SK2 (Bharwad and Rajkumar, 2020).

Rhizosphere, the intended niche of the inoculated phosphate solubilizing bacteria (PSB), have plentiful mixtures of organic compounds like sugars, organic acids, and amino acids exuded by the roots (Kamilova et al., 2006). In the presence of multiple carbon sources, carbon catabolite repression (CCR) is plausible to run the preferential utilization of most efficient carbon source in rhizobacteria. Such preferences are important for the fitness of organisms in their natural environments for optimizing growth rate *via* controlling carbon flow. Though the mechanisms of CCR regulatory networks differ among bacterial species, many of these functions at the transcriptional or post-transcriptional level, controlling substrate uptake or expression of various catabolic enzymes (Rojo, 2010). The metabolically versatile pseudomonads use organic acids as the preferred carbon source over glucose. Succinate is known to inhibit the expression of many transporters and catabolic genes involved in the metabolism of several other carbon sources, for example, those required for the utilization of glucose and mannitol (MacGregor et al., 1991; Hester et al., 2000). The CCR regulatory network in pseudomonads is governed by several potential regulator proteins such as CyoB, PtsN, and PtsO and the Crc (catabolite repression control) protein (Görke and Stülke, 2008; Rojo, 2010).

Catabolite repression control protein was originally reported in *Pseudomonas aeruginosa*, and a mutation in the *gene* locus resulted in a strain that could utilize glucose and mannitol even in the presence of tricarboxylic acid (TCA) intermediates (MacGregor et al., 1991; Wolff et al., 1991). The protein sequence of Crc resembles nuclease family, though it did not show any nuclease or DNA-binding activity (MacGregor et al., 1996). The mechanism by which Crc regulates expression of carbohydrate metabolism remains unclear. The existing models in *P. aeruginosa*, *Pseudomonas putida*, and *Pseudomonas syringae* indicate that CCR takes place at the post-transcriptional level and arbitrate it to complex interactions among numerous regulatory components (Sonnleitner et al., 2009; Filiatrault et al., 2013; Hernández-Arranz et al., 2013). Besides Crc, another key player known for its regulatory role in overall bacterial physiology is the RNA chaperone Hfq (Vogel and Luisi, 2011). The Hfq protein was first found in *Escherichia coli* as a host factor required for replication of Qβ bacteriophage (thus regarded as Hfq) (Franze de Fernandez et al., 1968). Hfq is considered a global transcriptional controller participating in various cellular processes like outer membrane biogenesis, stress tolerance, virulence, and iron homeostasis (Chao and Vogel, 2010). The influence of Hfq on physiology and virulence in a number of Gram-negative and in Gram-positive bacteria has been reported (Simonsen et al., 2011; Wang et al., 2014; Kuo et al., 2017).

Regardless of the study of CCR in many gammaproteobacteria, attempts to unravel the key components and the molecular mechanism of CCR in bacteria other than members of the *Pseudomonas* group are limited. Zimmermann et al. (2009) identified that Crc protein is involved in the CCR in *Acinetobacter baylyi*. The key enzyme of protocatechuate degradation, protocatechuate 3,4-dioxygenase, was strongly reduced in the *crc*^–^ strain, while in the wild-type strain, it endured strong catabolite repression by acetate and succinate. Here, we describe CCR of the MPS phenotype in the plant growth-promoting bacteria *Acinetobacter* sp. SK2. This work specifically focuses on succinate-induced repression of *gdhA* and *gdhB* genes, causing diminished gluconate production. The role of Crc as putative regulator of *gdhA* and *gdhB* was investigated by generating insertionally inactivated mutants of Crc. Although *Acinetobacter* sp. SK2 is phylogenetically related to *Pseudomonas* species, it possesses a distinctive feature, i.e., the presence of sGDH, which has a role in glucose oxidation and was repressed in the presence of succinate (or along with glucose) similar to mGDH (Bharwad and Rajkumar, 2020). The glucose utilization and gluconate-mediated MPS of *Acinetobacter* sp. SK2 were investigated by functional characterization of the two key enzymes involved in glucose oxidation. To this purpose, the mGDH encoding gene *gdhA* and the sGDH encoding gene *gdhB* of *Acinetobacter* sp. SK2 were inactivated systematically. The physiological and metabolic attributes of the respective gene mutation on the MPS phenotype were investigated thereafter. The expression profile of Hfq in glucose and glucose + succinate opened another avenue of investigating Hfq as the next putative regulator of carbon source utilization along with the Crc in *Acinetobacter* sp. SK2.

## Materials and Methods

### Bacterial Strains, Plasmids, and Chemicals Used

The list of bacterial strains and plasmids used in the study is shown in Table 1. *Acinetobacter* sp. SK2 is a previously characterized mineral phosphate solubilizing isolate (Bharwad and Rajkumar, 2020). The suicide vector, pKnockoutΩ, was obtained from Dr. Filiatrault, Cornell University (Windgassen et al., 2000). *E. coli* DH5α was used to harbor the native suicide vector and the newly constructed knockout plasmids. Antibiotics added to the medium, for selection and maintenance of the plasmid, were in the following concentration unless otherwise mentioned: 100 μg ml^–1^ of ampicillin, 50 μg ml^–1^ of streptomycin, and 50 μg ml^–1^ of spectinomycin. For the post-pulse recovery of electoporated *Acinetobacter* cells, super optimal broth (SOC) medium was used. All the plasmids were isolated using HiPurA Plasmid DNA miniprep purification kit (HiMedia, Mumbai, India) and adjusted to the final concentration of 50 ng μl^–1^.

**TABLE 1:** Bacterial strains and plasmids used in the study.

**Strain or plasmid**	**Relevant characteristics**	**Origin/references**
*Acinetobacter* sp. SK2	P solubilizing isolate, Host, Wild-type (GenBank accession number MG847098)	Bharwad and Rajkumar, 2020
*E. coli* DH5α	Host, ΔlacU169 (ϕ80lacZΔM15) recA1	Grant et al., 1990
pKnockoutΩ	Sm^r^ Sp^r^*mob* in T-vector	Windgassen et al., 2000
pKO*gdhA*	500bp *gdhA* fragment in pKnockoutΩ between *Kpn*I and *Xba*I sites	This study
pKO*gdhB*	550bp *gdhB* fragment in pKnockoutΩ between *Kpn*I and *Sal*I sites	This study
pKO*crc*	650bp *crc* fragment in pKnockoutΩ between *Sal*I and *Pst*I sites	This study

### Construction and Confirmation of Knockout Plasmids

*Acinetobacter* sp. SK2 mutants were constructed using suicide vector pKnockoutΩ. Linearized vector was generated using the three combinations of restriction enzyme; (1) *Kpn*I + *Xba*I, (2) *Sal*I + *Pst*I, and (3) *Kpn*I + *Sal*I for insertion of *crc*, *gdhA*, and *gdhB*, respectively. The 650-bp *crc*, 500-bp *gdhA*, and 550-bp *gdhB* insert fragments were amplified from chromosomal DNA of *Acinetobacter* sp. SK2 using oligonucleotide primers described in Table 2. In-Fusion HD Plus Cloning kit (Takara Bio Inc., Kusatsu, Shiga, Japan) was used for the ligation of the linearized vector with the insert fragment, and the ligation mixture was used for transformation of the competent *E. coli* DH5α cells. The recombinant clones were selected through blue-white screening on Luria–Bertani (LB) agar containing ampicillin, streptomycin, and spectinomycin. Digestion of plasmids isolated from the recombinant white colonies was performed using restriction enzyme combinations of the respective gene inserts. Insert release of 650, 500, and 550 bp from the *crc*, *gdhA*, and *gdhB* knockout constructs confirmed a successful insertion of desired gene into the knockout vector. These constructs were named pKO*crc*, pKO*gdhA*, and pKO*gdhB*, respectively, and were used for electrotransformation of *Acinetobacter* sp. SK2.

**TABLE 2:** Primers used in the study.

**Primers for qPCR**	
Primers	Relevant characteristics (5′→3′)
Hfq-qF	AAAGCCGCTACCTTGACCAC
Hfq-qR	TCCAGCACGTAATCCACGTC
Crc-qF	TCTCTTTCCAGCAGAGCGTG
Crc-qR	GTCAGCCAGCTCAAACCCTA
GdhA-qF	TCAACACCATACGGTACGCC
GdhA-qR	CCTGATCGTCAGGTTGCTGT
GdhB-qF	ACCCCTGCGGCTACTTTTAC
GdhB-qR	ATGGTTGGCCGAATGTAGCA
RpoD-qF	TCAGGCTCGCACAATTCGTA
RpoD-qR	TGGTTCACGGCCCATTTTCTT
**Primers for in-fusion Cloning and confirmation of mutation**	
CINF	**TATAGGGCGAATTG**GGTACCTACGTTCTT CAGTAACGAAAGGCT with *Kpn*I underlined site
CINR	**TGGCGGCCGC**TCTAGACACAAATGATGAC TGACTTGTTTTC with *Xba*I underlined site
SINF	**TATAGGGCGAATTG**GGTACCCTGATGTTCC TCTAATTCCATCTC with *Kpn*I underlined site
SINR	**TATCGATACC**GTCGACTCACCAATCGTAT AATAAATCTTTT with *Sal*I underlined site
MINF	**CCCCCTCGA**GTCGACTACTGGCGTTATTCC TGTTAATTGG with *Sal*I underlined site
MINR	**CATGTCATGACTGG**CTGCAGTGAGTACGACC ATAAGCTGGCC with *Pst*I underlined site
Omega	ATGATCAGCCGAGATAGCA

*The bold sequence at 5′ end denotes homologous region to vector. Restriction sites added at the 5′ end of primer are underlined (after homologous region to vector). MINF-Omega combination was used for confirmation of insertion of omega in *gdhA* gene. Likewise, the combination of SINF-Omega and CINF-Omega was used for verification of insertion of omega in *gdhB* and *crc*, respectively.*

### Electrotransformation of *Acinetobacter* sp. SK2

Members of the genera *Acinetobacter* are naturally competent and can take up high-molecular-weight extracellular DNA from a source in the natural environments (Bruns et al., 1992). However, to increase the transformation efficiency, electrotransformation was employed (Lorenz et al., 1992; Yildirim et al., 2016), in which 200 μl of overnight grown cells was transferred into 5 ml of fresh mineral medium supplied with 10 mM of succinate and incubated for 2 h at 37°C. In sterile, ice-cold electroporation cuvettes (Eppendorf, Hamburg, Germany), 500 μl of these cells was added with ∼100–250 ng of recombinant plasmids (constructs: pKO*crc*, pKO*gdhA*, and pKO*gdhB*) as individual treatments. All electroporations were conducted in Eporator (Eppendorf), set at 1.8 kV for 3 s to transform DNA into the bacterial cells. The electroporated cells were added to a fresh, sterile microfuge tube containing 500 μl of SOC medium followed by 2 h incubation at 37°C. For the selection of tranformants, 100 μl of cell culture was spread on LB medium supplemented with streptomycin and spectinomycin and was incubated at 37°C. Colonies were transferred on fresh selective media plates several times to ensure a pure culture.

### Confirmation of Insertion of Suicide Vector in *crc*^–^, *gdhA*^–^, and *gdhB*^–^ Strains

The *gdhA* mutants were spot inoculated on YGC medium (yeast–glucose–calcium carbonate medium containing g L^–1^: yeast extract 20; glucose 50; CaCO_3_ 10; and agar power 15) (Zhu et al., 2011) to observe phenotypic change caused by the inactivation of the gene. However, since no known visual phenotypic character of *crc* and *gdhB* is known, only molecular methods were used for confirmation of the successful gene disruption. The presence of omega (Ω) cassette in the genome of clones was confirmed through a colony PCR using oligonucleotide primer (Table 2). Integration of omega cassette into the correct chromosomal location was verified by PCR using a set of primers hybridizing the gene of interest and the omega cassette (Table 2). An increase in the amplicon size was indicative of the successful insertion of the omega cassette into the gene of interest, which was further confirmed by DNA sequencing.

### Growth Conditions

Strains of *Acinetobacter* sp. SK2 were grown on LB medium at 37°C on an orbital shaker incubator (Rojas-Tapias et al., 2014). For the cell growth experiments, enzyme assays, and expression analysis, all the strains were grown under identical conditions in M9 minimal medium at 37°C with a continuous agitation (Mara et al., 2012), where the carbon sources added individually were at the following final concentrations unless otherwise indicated: succinate, 50 mM; glucose, 50 mM; and glucose + succinate, 25 mM each. Specifications pertaining to preparation of cells for enzyme assay are described in the following sections. To ensure chromosomal integration of the antibiotic-encoding omega cassette, the cells were grown in the absence of antibiotics to ensure optimal growth, after which inoculation on the media containing the appropriate antibiotics was carried out to check the presence of the resistance omega cassette. Instability of the chromosomally integrated resistance cassettes was not observed. *E. coli* transformed with plasmid constructs were grown in LB medium at 37°C, supplemented with ampicillin (100 μg ml^–1^), streptomycin (50 μg ml^–1^), and spectinomycin (50 μg ml^–1^).

### Enzyme Activities of Membrane Glucose Dehydrogenase and Soluble Glucose Dehydrogenase

Glucose dehydrogenase activity in the wild-type and mutants was done using a chromogenic assay involving 2,6-dichlorophenolindophenol (DCIP; HiMedia) and phenazine methosulfate (PMS; SRL Chemicals, Mumbai, India). Cells were grown in M9 minimal medium with 50 mM of glucose; 50 mM of succinate; and 25 mM glucose + 25 mM succinate. For mGDH, a whole cell pellet was obtained through centrifugation at 12,000 *g* at 4°C for 5 min. The pellet was resuspended in 50 mM of potassium phosphate buffer (pH = 7.5) with MgCl_2_. For assay of the sGDH, the cell pellet was suspended in a small volume of same buffer containing 20% glycerol and 1 mM of DTT and sonicated for 5 min at a pulse rate of 30 s at 50 Hz on ice. The suspension was centrifuged at 11,000 *g* at 4°C for 30 min in order to remove cell debris. Furthermore, to separate out the crude soluble fraction, the supernatant was subjected to ultracentrifugation at 120,000 *g* for 90 min and was immediately used for the sGDH enzyme assay (Matsushita et al., 1989).

### Expression Analysis of *crc*, *gdhA*, and *gdhB* Genes in Wild Type and Mutants

The relative expression of genes was estimated using quantitative real-time PCR (qRT-PCR). cDNA was synthesized from RNA isolated from the wild type and mutants grown in M9 minimal medium containing glucose, succinate, and glucose + succinate. Cells were harvested at different growth stages and subjected to total RNA isolation using Hybrid-R^TM^ Kit (GeneAll^®^), as per manufacturer’s instructions. Residual genomic DNA contamination was removed using DNase I (Thermo Fisher Scientific, Waltham, MA, United States) followed by DNase inactivation by adding 2 μl of stop solution containing EDTA and heating at 65°C for 10 min. Finally, the DNA contamination was checked by PCR using 16S rRNA universal primers F27 (5′-AGAGTTTGATCATGGCTCAG-3′) and R1492 (5′-TACGGTTACCTTGTTACGACTT-3′), and the RNA integrity was determined on agarose gel electrophoresis. cDNAs from each sample were obtained using PrimeScript^TM^ 1st strand cDNA synthesis kit (Takara Bio Inc.) following the manufacturer’s instructions. qPCR was performed on an Applied Biosystems QuantStudio4 system using a SYBR Premix Ex Taq II (Tli RNaseH Plus) kit (Takara Bio Inc.). Amplification and detection of the specific products were performed with three technical replicates per sample under the following conditions: one cycle at 95°C for 30 s, followed by 40 cycles of denaturation at 95°C for 3 min, and annealing and extension at 60°C for 1 min. Cycle threshold (Ct or Cq value) values were recorded with QuantStudio^TM^ Design and Analysis Software (Thermo Fisher Scientific). Ct values of 35 were considered beyond the limit of detection (Chen et al., 2009). Fold changes were determined using the relative quantification comparative threshold cycle (Ct) method (DDCt) (Livak and Schmittgen, 2001), and *rpoD* housekeeping gene was used as a reference for normalization. Oligonucleotide primers used for the qRT-PCR are shown in Table 2. For each gene tested, a standard curve was plotted using serial dilutions from 50 to 0.005 ng of *Acinetobacter* sp. SK2 genomic DNA in order to quantify the efficiency of each PCR. The PCR products ranged between 100 and 150 bp.

### Effect of Insertional Inactivation on Mineral Phosphate Solubilization Phenotype

Qualitative estimation of MPS in the wild type and mutants was performed on Pikovskaya’s agar (Pikovskaya, 1948) and Tris-buffered rock phosphate (TRP) agar, as described by Rajput et al. (2013). Quantitative estimation of soluble P was carried out in Pikovskaya’s agar and TRP broth containing glucose (50 mM), succinate (50 mM), or equimolar mixture of glucose + succinate (25 mM each; repression medium). P released in the medium was quantified by a reaction of soluble P with ammonium molybdate to form phosphomolybdate, which when reduced by ascorbic acid forms a blue colored complex that is quantified at 820 nm (Ames, 1966).

### Effect of Wild-Type and Mutant *Acinetobacter* sp. SK2 on Growth of *Vigna radiata*

Plant growth experiments were carried out in pots sown with mung bean (*Vigna radiata*) as reported by Kumari et al. (2018). Soil samples were collected from the Nirma herbal health garden, air dried, cleaned, and sterilized by three repeats of autoclave cycles. Soil was allowed to cool down, and 1 kg of soil was filled in each pot. *V. radiata* seeds were surface sterilized using 95% ethanol and then 0.2% HgCl_2_ surface sterilant for 3 min each. Sterilant was totally removed by washing seeds 10 times with the sterile distilled water. Eight to ten seeds were sown in each pot at a depth of 1–5 cm. Post germination, the plantlets were kept in sunlight and watered daily. Plantlets were inoculated with the wild-type, *gdhA*^–^, *gdhB*^–^, and *crc*^–^ strains (10^7^−10^8^ cfu ml^–1^) on the seventh day of germination, and the uninoculated plantlets served as a control. Plants were gently uprooted on the 31st day post germination and scored for plant parameters like root length (cm), shoot length (cm), root:shoot ratio, and dry mass (g).

## Results

### Generation of *gdhA*, *gdhB*, and *crc* Mutants of *Acinetobacter* sp. SK2

To evaluate the role of mGDH, sGDH, and Crc in the MPS of *Acinetobacter* sp. SK2, the individual mutant strains defective in *gdhA*, *gdhB*, and *crc* were constructed. Fragments of 500-bp *gdhA*, 540-bp *gdhB*, and 650-bp *crc* were cloned in a suicide vector pKnockoutΩ near the omega fragment (streptomycin–spectinomycin cassette). The resulting plasmids were transferred into the wild type *via* electroporation. As pKnockoutΩ cannot replicate in *Acinetobacter* sp. SK2, single-crossover integrants were selected by resistance to streptomycin–spectinomycin. An integrant lacking the *gdhA* that produced no or little gluconate on glucose was selected on YGC medium. The selected strains with insertional inactivation were named *gdhA*^–^, *gdhB*^–^, and *crc*^–^. The correctness of the insertion of the respective genes in the integrants was verified by appropriate PCR amplifications (Supplementary Figure 1). The vector construction and general strategy employed for mutant generation are described in Supplementary Figures 2, 3.

### P Solubilization by *gdhA*^–^, *gdhB*^–^, and *crc*^–^ Strains

**FIGURE 1:**
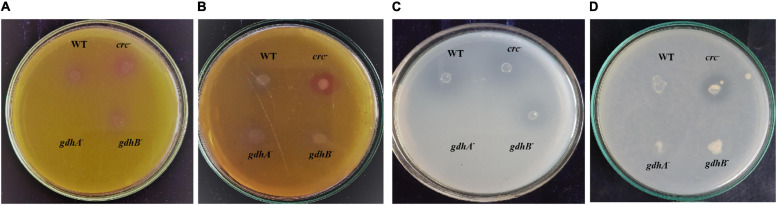
MPS phenotype of wild-type, *gdhA*^–^, *gdhB*^–^, and *crc*^–^ mutants on TRP agar **(A)** with glucose and **(B)** with glucose + succinate; Pikovskaya agar **(C)** with glucose and **(D)** with glucose + succinate.

The MPS ability of the wild type was significantly reduced in medium containing succinate. The addition of succinate to glucose-containing medium repressed the glucose utilization and oxidation to gluconate, which is achieved by the mGDH activity. The sGDH, along with mGDH, was also found to be under the repressing influence of succinate (Bharwad and Rajkumar, 2020). To evaluate the function of mGDH and sGDH in glucose oxidation and relevance of the Crc protein in this succinate-mediated repression, we compared the MPS ability of the wild type and the *gdhA*^–^, *gdhB*^–^, and *crc*^–^ strains on PVK and TRP media containing glucose, succinate, and glucose + succinate. The wild-type, *gdhB*^–^, and *crc*^–^ strains showed tricalcium phosphate (TCP) solubilization (halo zone around the colony) and rock phosphate (RP) (pink coloration around the colony) in the presence of glucose; however, *gdhA*^–^ failed to grow on the media (Figures 1A,C). The wild-type, *gdhA*^–^, and *gdhB*^–^ failed in MPS on glucose + succinate, while the phenotype was observed in *crc*^–^ (Figures 1B,D).

The soluble P released by the wild-type and mutant strains in PVK and TRP broth media supplemented with glucose, succinate, and glucose + succinate in the treatments is presented (Table 3). The free P measured in succinate and glucose + succinate in all the treatments was comparable with the uninoculated medium (pH 7 or 8) or the soluble P measured on day 0. The maximum P released by the wild type in the media containing glucose + succinate was only 7% of the P released in the glucose-containing media. The repression of P solubilization in PVK medium was 90–91% in glucose + succinate- and succinate-containing media. The amount of soluble P released during the course of growth in glucose + succinate and succinate was insignificant. A similar trend was observed for RP solubilization where soluble P decreased up to 97–99% in glucose + succinate and succinate (Table 3).

**TABLE 3:** Phosphate solubilization by *Acinetobacter* sp. SK2 wild-type, *gdhA*^–^, *gdhB*^–^, and *crc*^–^ strains in PVK and TRP broth medium.

	Soluble P release and acidification in PVK medium* (initial pH-7)				Soluble P release and acidification of TRP medium* (initial pH-8)			
**Carbon source**	**Wild-type SK2**	***gdhA*^–^**	***gdhB*^–^**	***crc*^–^**	**Wild-type SK2**	***gdhA*^–^**	***gdhB*^–^**	***crc*^–^**
Glucose	688.7 ± 0.21 (4.0 ± 1.0)	60.7 ± 3.9 (6.5 ± 1.0)	603.3 ± 15.3 (3.9 ± 0.2)	550.9 ± 10.6 (4.3 ± 0.2)	86.3 ± 9.0 (4.6 ± 0.8)	4.3 ± 1.6 (8.2 ± 0.3)	83.2 ± 8.4 (3.7 ± 0.4)	42.7 ± 2.5 (4.1 ± 0.1)
Glucose + succinate	60.7 ± 3.9 (7.9 ± 0.1)	62.9 ± 6.9 (6.0 ± 0.4)	63.0 ± 2.6 (7.7 ± 0.2)	445.1 ± 9.1 (4.2 ± 0.2)	3.2 ± 1.7 (8.0 ± 0.1)	0.7 ± 0.1 (8.2 ± 0.1)	5.3 ± 2.1 (8.1 ± 0.1)	18.0 ± 2.6 (8.3 ± 0.3)
Succinate	65.0 ± 8.7 (8.1 ± 0.2)	62.9 ± 6.9 (6.8 ± 0.1)	63.2 ± 6.7 (7.9 ± 0.1)	65.0 ± 8.7 (8.6 ± 0.3)	1.4 ± 0.1 (8.6 ± 0.1)	0.7 ± 0.2 (8.6 ± 0.1)	1.7 ± 1.2 (8.6 ± 0.3)	0.4 ± 0.2 (8.4 ± 0.2)

*The soluble P released (μg ml^–1^) from (TCP) and (RP) and pH measured on day 3. The values in the brackets indicate the pH. Values are mean ± standard deviation of three independent observations in triplicates.

The P release was almost completely abolished in *gdhA*^–^ as compared with the wild type. The amount of P released by the mutant was similar to that of the wild-type in glucose + succinate- and succinate-containing media. The absence of *gdhA* and abolition of MPS also correlated to the pH of the medium that remained unchanged.

The P release and its repression in *gdhB*^–^ in glucose + succinate were similar to those of the wild type. Disruption of *gdhB*^–^ influenced neither P solubilization nor mGDH-mediated gluconate production. Likewise, the P released by *crc*^–^ strain in PVK was measured in medium supplemented with glucose, succinate, and glucose + succinate. The P release in the glucose media was 550 μg ml^–1^, which was similar to that of the wild type grown in glucose. An enhanced P release was observed in glucose + succinate medium, reaching up to 44% of the wild-type grown in glucose, indicating derepression. A concomitant decrease in pH up to 4.3 was the indication of acidification of the medium and P release in glucose + succinate media. It can therefore be concluded that *gdhA* was solely responsible for periplasmic oxidation of glucose to gluconate, which leads to the MPS (Table 3). When RP solubilization by *gdhA*^–^, *gdhB*^–^, and *crc*^–^ was compared with the wild type in TRP medium, a trend similar to TCP solubilization was observed (Table 3).

### Changes in Growth Profiles of *gdhA*^–^, *gdhB*^–^, and *crc*^–^ Strains

Since the wild type preferentially utilized succinate over glucose in medium containing glucose + succinate affecting gluconate-mediated MPS phenotype (Bharwad and Rajkumar, 2020), the differences in the growth of the three mutants in medium containing glucose, succinate, and glucose + succinate were observed.

**FIGURE 2:**
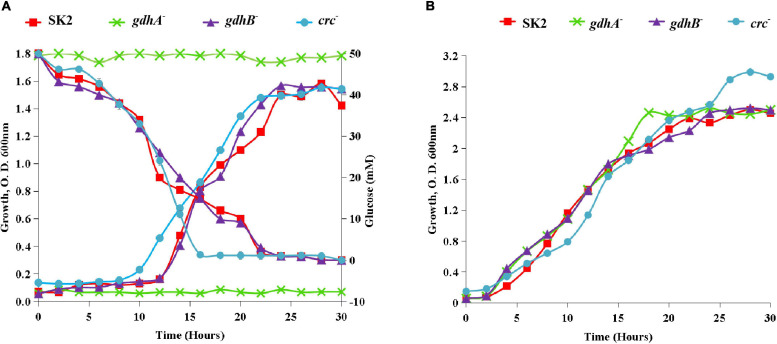
Monoauxic growth profile of wild-type SK2 and mutants on **(A)** glucose and **(B)** succinate. SK2 (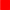), *gdhA*^–^ (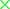), *gdhB*^–^ (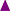), and *crc*^–^ (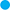).

Though there were no differences in the growth of the wild type, *gdhB*^–^, and *crc*^–^ on glucose, significant deviation in cell growth on glucose was observed in the case of *gdhA*^–^ strain (Figure 2A). This observation suggested that *gdhA* contributed mostly to glucose utilization or oxidation in the wild type. The inactivation of sGDH encoding gene *gdhB* did not influence the growth of the mutant on glucose. The growth of all the three mutants in succinate was similar to that of the wild type (Figure 2B). The wild-type and *crc*^–^ strains took 20 h for the complete glucose consumption, while *gdhB*^–^ required 15 h to completely exhaust glucose from the medium. All the strains grew on glucose after a lag phase of 8–10 h, the growth being slower and significantly lower than on succinate. A prolonged lag phase in glucose compared with succinate indicated that succinate was more rapidly utilized in the wild type (Figure 2).

**FIGURE 3:**
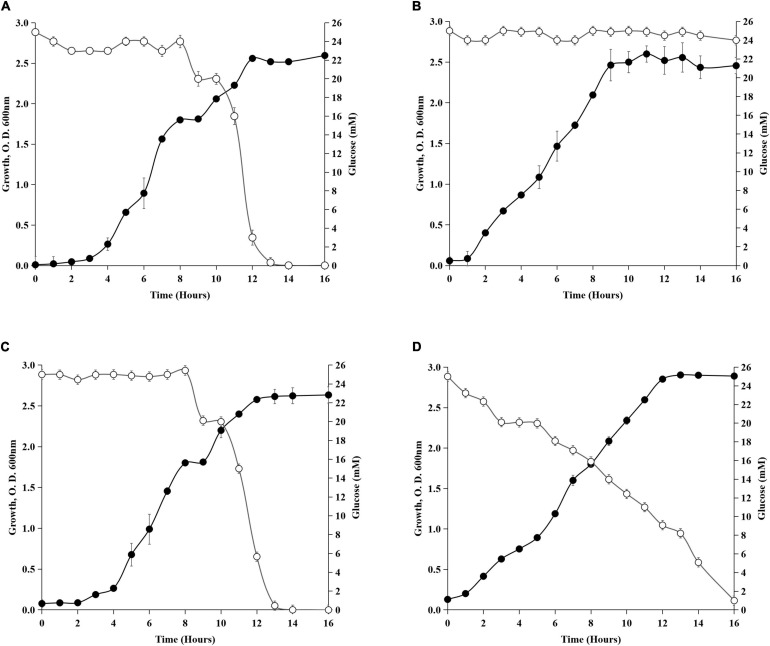
Diauxic growth profile on glucose + succinate. **(A)** Wild-type SK2, **(B)** *gdhA*^–^, **(C)** *gdhB*^–^, and **(D)** *crc*^–^. Filled circles represent the growth and open circles represent the glucose concentration (mM) in the medium.

Though the molecular mechanism of preferential utilization of succinate over glucose is not clear in *Acinetobacter* sp., similarities can be drawn from the earlier studies on gammaproteobacteria *Pseudomonas* sp. The intracellular phosphorylative pathway for glucose utilization was suppressed in *P. putida* CSV86 when grown in the presence of succinate (Basu et al., 2006). The glucose catabolic enzymes were found to be repressed by succinate in *Rhizobium* and *Bradyrhizobium* (Mandal and Chakrabartty, 1993). How the preferential utilization of succinate over glucose and absence of *gdhA*, *gdhB*, and *crc* influence growth of *Acinetobacter* sp. was investigated (Figure 3). The wild type showed diauxic growth curve on glucose + succinate (Figure 3A), which had a distinct first log, second lag, and second log phases. Succinate utilization during the first log phase was followed by glucose utilization, which was consumed after 7–8 h of incubation, when succinate was completely exhausted from the media. The diauxic growth of the wild type was absent in *gdhA*^–^. Though *gdhA*^–^ could not utilize glucose, it continued to grow due to presence of succinate in the medium (Figure 3B). The absence of *gdhA* gene or loss of mGDH caused no impairment in succinate uptake. The *gdhB*^–^ when grown on glucose + succinate had a profile similar to that of the wild type. This observation implied that the absence of *gdhB* might not have any major role in glucose uptake in the wild type (Figure 3C). In *crc*^–^, glucose utilization and growth started from the late lag phase and continued with gradual decrease of glucose from the medium throughout the growth phase. The loss of diauxie in *crc*^–^ confirmed that Crc was involved in regulating the preferential utilization of succinate over glucose, as *crc*^–^ continued utilization of glucose regardless of the presence of succinate (Figure 3D).

### Alteration of GDH Activity in *gdhA*^–^, *gdhB*^–^, and *crc*^–^ Strains

**FIGURE 4:**
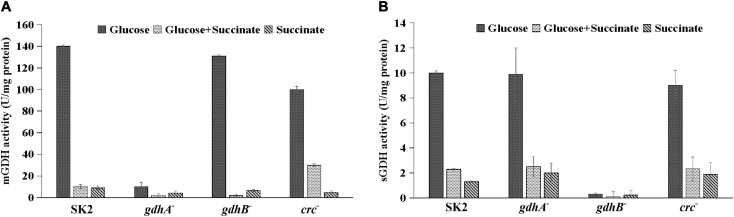
Enzyme activity of glucose, glucose + succinate, and succinate-grown *Acinetobacter* sp. SK2, *gdhA*^–^, *gdhB*^–^, and *crc*^–^ **(A)** membrane glucose dehydrogenase(mGDH) and **(B)** soluble glucose dehydrogenase (sGDH).

To correlate the biochemical functions of the *gdhA* and *gdhB* genes, enzyme activities of mGDH and sGDH were assayed in the crude cell extracts of the wild-type and mutant strains grown in glucose, succinate, and glucose + succinate-containing media (Figure 4). Since succinate is not a substrate of GDH, no or insignificant mGDH or sGDH activity was observed in the wild-type or mutant strains as expected. In contrast to the high activity of the wild type in glucose, the mGDH activity of *gdhA*^–^ was abolished (Figure 4A). The mGDH activity of *gdhB*^–^ remained similar to that of the wild type. Under repression conditions, i.e., in glucose + succinate, the mGDH activity was repressed in the wild type as well as in *gdhA*^–^ and *gdhB*^–^. To assess the involvement of the Crc protein in repression, the GDH activities in the wild-type and the *crc*^–^ strains were compared. The *crc* inactivation resulted in an increase of mGDH activity by 30% in glucose + succinate-grown *crc*^–^ cells as compared with the wild type, indicating derepression. The mGDH and sGDH activities of succinate-grown wild-type cells were only 4–6% of those of the glucose-grown cells. The sGDH activity was completely abolished in glucose-grown *gdhB*^–^, which remained unaltered in *gdhA*^–^ and *crc*^–^ (Figure 4B). The sGDH activity in glucose + succinate-grown cells of *gdhA*^–^ and *crc*^–^ was repressed to the extent of the wild type. The results indicated that the *gdhA* and *gdhB* genes in *Acinetobacter* sp. SK2 encoded enzymes functionally equal to the mGDH and sGDH, respectively.

### Quantitative Real-Time PCR Analysis of Glucose Oxidation and Carbon Catabolite Repression-Related Genes

**FIGURE 5:**
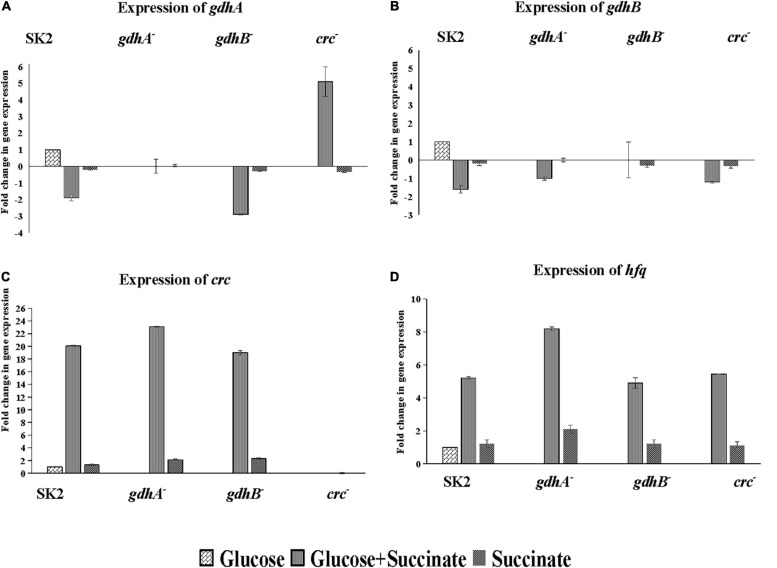
Relative expression of glucose oxidation and CCR genes in *Acinetobacter* sp. SK2 and mutants on glucose, succinate, and glucose + succinate. Expression of **(A)** *gdhA*, **(B)** *gdhB*, **(C)** *crc*, and **(D)** *hfq*. All cells grown to the exponential growth phase were harvested for total RNA isolation. Glucose grown wild-type cells were considered control for calculation of relative expression of all the genes in mutants.

Glucose and succinate are abundant in the rhizosphere; however, the preference among the two will differ among the different groups of bacteria. Therefore, it is probable that the expression of genes required for metabolism of non-preferred carbon source is subjected to repression by the preferred carbon substrates. The nature of CCR has not been examined in *Acinetobacter* while mimicking the rhizosphere conditions in context to sugars (glucose) and organic acids (succinate). To this end, insertional mutants of glucose oxidation and CCR-related genes were generated. The transcriptional profiles of *gdhA*, *gdhB*, *crc*, and *hfq* were measured in cells grown on glucose, succinate, and glucose + succinate. Gene transcriptions in the glucose-grown cells were used as a control. The data acquired indicated strong inhibition of *gdhA* and *gdhB* by succinate in the wild type (Figures 5A,B). High transcriptional levels of *gdhA* in glucose (Figure 5A) along with the high enzyme activity (Figure 4A) supported that glucose oxidation was catalyzed by the *gdhA* gene encoding mGDH enzyme in the wild type. A 1.9-fold downregulation of *gdhA* in the repression medium was an evidence of succinate-mediated catabolite repression (SMCR) of mGDH in the wild-type strain. A significant reduction of *gdhA* expression was observed in the presence of glucose + succinate in the wild-type but not in the *crc*^–^ strain (Figure 5A). These data clearly indicate relaxation of succinate-provoked CCR in *crc*^–^ strain, which was supported by soluble P estimation (Table 3) and enzyme assay data (Figure 4A) in repression condition. Surprisingly, the expression and activity of *gdhA* in *gdhB*^–^ remained unaltered (Figure 5A). Notably, a more pronounced SMCR was detected in the wild type, and the inactivation of *crc* caused a significant increase in phosphate solubilization (Table 3) and mGDH activity (Figure 4A) under the CCR conditions. However, as expected, insertional inactivation of *crc* did not abolish the CCR completely, which entails the participation of other yet-unknown CCR regulators.

The expression of *gdhB* in all the mutants and wild type was downregulated irrespective of the genes mutated with the fold change of 1.2–1.6 (Figure 5B). No expression of *gdhA* and *gdhB* in both *gdhA*^–^ and *gdhB*^–^ grown in succinate was in line with the enzyme activity (Figure 4).

The expression of *crc* gene was induced in the CCR condition, i.e., in glucose + succinate medium in the wild type as well as in *gdhA*^–^ and *gdhB*^–^. Since *crc* was expressed only in the presence of secondary carbon source, it is implicated in CCR of the MPS (Figure 5C), and its mutation increased the mGDH activity (Figure 4A). A similar trend in expression of *hfq* gene in the cells grown in glucose + succinate was observed with lower fold changes as compared with *crc* (Figure 5D). Succinate being the preferred carbon source induced a basal expression of *crc* in the wild type, *gdhA*^–^, and *gdhB*^–^ (Figure 5C), while the expression of *hfq* was recorded in the wild type and the three mutants (Figure 5D).

### Effect of Wild-Type and Mutant Strains on the Growth of *Vigna radiata* Plant

Since phosphate solubilization is one of the important plant growth-promoting traits in the bacteria used as PGPR, the ability of the wild type and mutants in promoting growth of *V. radiata* was tested. *V. radiata* inoculated with bacteria were monitored for plant growth parameters in a pot experiment (Table 4). Plants inoculated with the wild-type, *gdhA*^–^, *gdhB*^–^, and *crc*^–^ increased the root length and shoot length when compared with the control. The growth parameters in *crc*^–^ treatments were significantly higher compared with those of the wild type, *gdhA*^–^, and *gdhB*^–^. *crc*^–^ treatment significantly improved the root length and shoot length. An increased root length of ∼1.3-fold and shoot length of ∼1.4-fold was recorded in the plants inoculated with the wild type, *gdhA*^–^, and *gdhB*^–^. The *crc*^–^ treatment increased the root length by ∼1.5-fold and the shoot length by ∼1.4-fold compared with the uninoculated control. The treatment of plants with the wild-type, *gdhA*^–^, *gdhB*^–^, and *crc*^–^ significantly increased the fresh weight (∼1.2- to 1.6-fold) and dry mass (∼1- to 1.8-fold).

**TABLE 4:** Growth parameters of *Vigna radiata* inoculated with *Acinetobacter* sp. SK2 and mutant strains.

	Root length (cm)*	Shoot length (cm)*	Dry mass (mg)*	Fresh mass (mg)*
Control	9.50.5	21.40.8	872.4	30020.1
Wild-type SK2	12.30.5	28.21.6	1102.8	58910.1
*gdhA*^–^	13.20.3	24.51.3	921.4	3209.4
*gdhB*^–^	12.01.7	25.81.3	932.8	31212.0
*crc*^–^	14.51.3	30.20.9	1032.5	50924.0

*Significant as compared with control (*P* ≤ 0.05). Values for root length, shoot length, and dry mass are mean ± standard deviation of 10 plants (*n* = 10).

## Discussion

The pyrroloquinoline quinone (PQQ)-dependent GDH has been reported in a wide variety of bacterial species, for example, *Acinetobacter calcoaceticus*, *Gluconobacter suboxydans*, and *P. aeruginosa*, which can carry out periplasmic glucose oxidation. GDH belongs to the largest group of quinoproteins, which require redox cofactor PQQ for its activity (Duine et al., 1982). *In vitro* studies have shown that the GDH has binding sites for Mg^+2^; and *in vivo*, it can bind to Ca^+2^, PQQ, and especially the substrate glucose (Matsushita et al., 1989). Two types of GDH have been reported based on their localization within the cell. The membrane-bound GDH (mGDH or GDH A) has been reported in many Gram-negative bacteria like *Gluconobacter*, *Pseudomonas* (An and Moe, 2016; Suleman et al., 2018), and *Acinetobacter* species (Rasul et al., 2019); however, sGDH is less common. The atypical existence of sGDH or GDHB is unique to *Acinetobacter* sp. (Duine et al., 1982; Cleton-Jansen et al., 1988; Matsushita et al., 1995). This atypical character is used as a taxonomical marker for the characterization of *A. calcoaceticus*. The sGDH of *A. calcoaceticus* is a homodimer consisting of identical subunits of 55 kDa, each binding to one molecule of PQQ. The amino acid sequences among the mGDH and sGDH are homologous only at the PQQ binding C-terminal region of the protein (Duine et al., 1982; Matsushita et al., 1989). Furthermore, the mechanistic properties of sGDH and mGDH from *A. calcoaceticus* are shown to be distinct. Since both sGDH and mGDH catalyze similar reactions involving same prosthetic group, their role in glucose oxidation, gluconate-mediated MPS, and its succinate-mediated repression was reported (Bharwad and Rajkumar, 2020). It was hypothesized that the Crc protein, which is known to govern carbon source utilization in *Pseudomonas* and related species, has a similar role in *Acinetobacter* sp. SK2. The putative role of Crc in regulating in the SMCR of the MPS phenotype was investigated by generating insertionally inactivated mutants of *crc* (Figure 1). The genome sequence of *Acinetobacter pittii* PHAE2 was used to generate mutants of *gdhA*, *gdhB*, and *crc* because its 16S rDNA sequence was 98.42%, similar to that of *Acinetobacter* sp. SK2.

In this study, the mutant strains of mGDH and sGDH were constructed to determine the role of GDHs in the MPS of *Acinetobacter* sp. SK2 (Supplementary Figures 1–3). It was evident from the growth profile that *gdhA* inactivation (*gdhA*^–^) abolished glucose oxidation as glucose remained in the spent medium (Figure 2A). The growth profile of *gdhA*^–^ mutants in succinate was similar to that of the wild-type strain (Figure 2B). The diauxic growth and glucose utilization pattern in glucose + succinate that indicated preferential utilization of succinate over glucose was also identical to that of the wild type (Figure 3). These results are in line with those of *Pseudomonas* sp. where organic acids have been reported to suppress the uptake and activity of glucose-catabolizing enzymes (Rojo, 2010). Repression of phenanthrene degradation in *P. putida* by the plant root extract and exudates containing glucose, acetate, and amino acids has also been reported (Rentz et al., 2004).

The complete absence of mGDH activity (Figure 4) and P release was also evident in the *gdhA*^–^ mutant; and therefore, it was concluded that the mGDH was solely responsible for gluconate production and MPS (Table 3). Similar results were obtained in *Gluconobacter oxydans*, which is broadly used for various biotechnological applications. In a previous study, to achieve improved growth properties on glucose, a *gdhA*^–^ strain was constructed, which led to loss of periplasmic glucose oxidation and gluconate production (Zhu et al., 2011). Deletion of GDH in *Serratia marcescens* resulted in loss of acidification of the medium, which confirmed its role in gluconate production (Fender et al., 2012). In this study, *gdhA* inactivation completely abolished the MPS and glucose utilization throughout the growth (Figures 1, 3). It is assumed that the wild-type cells might be converting total glucose into gluconate, which is further internalized by the cell for growth when glucose is the sole carbon source. It is possible that most of the glucose available to the cell (20 out of 50 mM of glucose) in the medium was used for MPS (Bharwad and Rajkumar, 2020). These results were in correlation with those of Gyaneshwar et al. (1999) where MPS mutants of *Enterobacter asburiae* deficient in the GDH activity failed to release the phosphate from alkaline soils, indicating that the GDH activity was required to release the soluble P.

The inactivation of *gdhB* did not alter the growth of cells in glucose or glucose + succinate when compared with the wild type (Figure 2). Absence of sGDH activity in glucose confirmed *gdhB* disruption (Figure 4B); however, the unaltered levels of P released or pH confirmed that the extracellular oxidation of glucose to gluconate did not employ intracellular glucose dehydrogenation by sGDH. The gluconate produced and detected in high-performance liquid chromatography (HPLC) analysis (Bharwad and Rajkumar, 2020) was solely due to the activity of mGDH. Cleton-Jansen et al. (1988) showed the presence of a second glucose dehydrogenase apart from mGDH and explained the difference between the *in vivo* and *in vitro* substrate specification. The soluble and membrane-bound enzymes have different properties including molecular size, substrate specificity, and kinetics in *A. calcoaceticus* LMD 79.41 (Matsushita et al., 1988). sGDH is currently used in the accurate monitoring of blood glucose using diabetic control test strips (Yamazaki et al., 2008). Being different from mGDH, sGDH was postulated to be involved in an electron transport to glucose oxidation, but a mutant strain containing only sGDH did not show any *in vivo* activity of glucose oxidation (Cleton-Jansen et al., 1988). In this study, *gdhB*^–^ did not show sGDH activity in glucose-containing medium; therefore, it was concluded that *gdhB* was an indispensable gene for sGDH activity and the oxidation of glucose leading to P solubilization did not employ sGDH. sGDH has been reported to bind PQQ rapidly, with high affinity and with larger conformational changes than mGDH (Cleton-Jansen et al., 1988; Matsushita et al., 1995). Therefore, it was proposed by Matsushita et al. (1995) that the function of sGDH could be of a PQQ carrier protein. sGDH is situated in the cytoplasm, and it could be accumulating PQQ from the external or internal milieu of the cells and may transfer PQQ to the target mGDH situated on the outer membrane to make it functional. Certainly, this was one of the assumptions made for the physiological role of sGDH, but more supporting experimental evidences are required for verification of the function of sGDH, as *gdhB* inactivation had no influence on the mGDH activity (Figure 4A). Wei et al. (2014) reported similar results upon deletion of sGDH gene in *G. oxydans* 621H. However, these results were contradictory from those reported for *G. oxydans* N44-1 where an improved cell growth rate was observed in glucose with the *gdhA* and *gdhB* double-gene knockout strain in comparison with the mGDH single-gene-deficient strain. The growth results of *G. oxydans* N44-1 double-gene deletion strain also suggested that glucose metabolism in this strain was not limited by the deficiency of the sGDH gene (Krajewski et al., 2010). Furthermore, the MPS of the *gdhB*^–^ in glucose was similar to the P release of the wild type (Table 3). This disproved that sGDH had a role in extracellular glucose oxidation, gluconate secretion, or the MPS phenotype.

Several lines of evidence suggest that Crc acts post-transcriptionally in many Gram-negative bacteria like *P. aeruginosa* and *P. putida* to regulate the repressed utilization of polycyclic aromatic hydrocarbons (PAHs) in the presence of succinate (Hester et al., 2000; Yuste and Rojo, 2001). The deletion of *crc* in *A. baylyi* did not alter the growth on a number of carbon sources. However, the repression caused on enzyme of protocatechuate breakdown in succinate and acetate was strongly reduced in the *crc* deficient strain (Zimmermann et al., 2009). Therefore, it was hypothesized that the succinate-mediated repression of mGDH and possibly sGDH leading to the MPS phenotype was under the control of Crc, which upon inactivation may lead to derepression of the MPS phenotype. To assess the hypothesis, the Crc encoding gene *crc* was disrupted, and its impact on the MPS phenotype was explored. The growth of *crc*^–^ cells on glucose + succinate was similar to that of the wild type (Figure 1), with loss of diauxie in the repression medium (Figure 3). Glucose utilization from the lag phase of the growth indicated co-utilization of glucose and succinate. Being a global regulator, hindrance in cell growth could be expected upon *crc* disruption; however, no such negative influence on the growth of the cells was recorded. The mGDH activity of *crc*^–^ in succinate-containing medium was derepressed up to 40% (Figure 4A), while the sGDH activity remained unaltered. The derepression of mGDH activity increased MPS in the *crc*^–^ mutants under repression conditions (Table 3), which again indicated that mGDH was solely responsible for P solubilization, while sGDH had no significant contribution to the phenotype. This report confirms that Crc has a role in regulation of the succinate-mediated repression of mGDH and the MPS phenotype in *Acinetobacter* sp. SK2. The expression of *crc* varies according to growth conditions and carbon sources. In *Pseudomonas fluorescens* SBW25, “xylose + succinate” created CCR condition, while xylose and glycerol were co-utilized. The expression of *crc* was higher only in the CCR condition, whereas when the cells were shifted to non-CCR condition, the expression level of *crc* decreased, implicating that Crc was one of the top candidates of the CCR regulatory hierarchy for the perception of nutrient signals (Liu et al., 2017). In *P. aeruginosa*, the inactivation of *crc* reportedly modified the expression of at least 57 genes when grown in LB medium, whereas in basal salt medium with succinate, the expression of 95 transcripts was altered. Among these genes, many were involved in the transport and assimilation of sugars and amino acids (Sonnleitner et al., 2012). The Crc-mediated regulation is not limited to carbon metabolism and may influence several other properties. In *P. aeruginosa*, Crc regulates the Lon protease for rhamnolipid production and *rhl* quorum sensing (Yang et al., 2015), and its role has also been implicated in virulence, biofilm formation, and antibiotic resistance (O’Toole et al., 2000; Yeung et al., 2011).

Apart from Crc, Hfq (RNA chaperone) is involved in the riboregulation of diverse metabolic genes *via* Crc-small RNAs. An attempt to explore the differences in *hfq* expression during the CCR conditions was also made in this study. It was observed that the expression pattern of *hfq* was similar to that of the *crc*. Apparent expression of *hfq* in glucose + succinate (the CCR condition) suggested its involvement in carbon source uptake and metabolism (Figure 5). As Crc functions to bring down catabolism of non-preferred carbon sources in the presence of preferred substrates (Sonnleitner et al., 2009; Rojo, 2010; Moreno et al., 2012), the catabolite repression in *P. putida* requires the Hfq along with the Crc protein. Crc and Hfq operate CCR networks by mediating efficient binding on “catabolite activity motifs” (CA-motifs; consensus AAnAAnAA) of RNAs required for the expression of the target carbon source (Madhushani et al., 2015; Moreno et al., 2015). In *P. putida* CF600, the dimethylphenol *dmp* system is required for the catabolism of phenol and (methyl) phenols, which requires Hfq and Crc for controlling DmpR levels *via* binding to the CA-motif of *dmpR*m RNA under the CCR conditions (Madhushani et al., 2015). Though the preliminary results implicate Hfq in the sequential utilization of carbon sources, the characterization of the detailed regulatory mechanisms of Hfq and Hfq-regulated pathways of *Acinetobacter* sp. SK2 needs experimental evidence for the idea to be substantiated.

The wild-type and mutant strains improved the plant growth parameters of *V. radiata* (Table 4). Significant improvement was observed in plants inoculated with *crc*^–^. Derepression of TCP solubilization by *crc*^–^ in glucose + succinate (Table 3) suggested secretion of gluconate by the mutant strain even under repression condition. Hence, the disruption of *crc* would likely help in better performance of PGP bacteria in the rhizosphere. The PSB like wild-type SK2 when applied to the field may face constrains due to the multi-tiered regulation by Hfq and Crc, which may influence their performance. Crc deficiency may relieve such bacteria from the metabolic struggle in the environment and help them execute the customized traits or functions.

## Conclusion

The natural habitat of *Acinetobacter* sp. SK2, a promising phosphate solubilizer is the rhizosphere, a nutritionally complex environment. The presence, composition, and abundance of various nutritional substrates change according to the stages of the plant development. When inoculated, it would require continuous changes in gene expression, particularly for those involved in phosphate acquisition. When the organism utilizes root exudates, CCR is most likely to operate due to the presence of glucose and succinate. CCR governed by the Crc protein and Hfq controls the availability of several enzymes and transporters involved in the assimilation of secondary carbon sources. Yet the regulation exerted by Crc on utilization of secondary substrates (for example, glucose, leading to the MPS phenotype) in *Acinetobacter* sp. had until now been unexplored. In this study, we explored the role of the MPS-mediating enzymes (mGDH and sGDH) and Crc in *Acinetobacter* sp. SK2. Loss of the MPS ability in *gdhA*^–^ confirmed the role of mGDH in periplasmic oxidation of glucose to gluconate. Disruption of *gdhB*^–^ led us to some speculations about the physiological functions of sGDH and unraveled its non-involvement in gluconate production or MPS. By adopting a combination of quantitative physiology experiments and transcriptional patterns of key genes in the wild-type, *crc*^–^, *gdhA*^–^, and *gdhB*^–^ strains, we demonstrate that Crc (directly or indirectly) orchestrates the MPS phenotype of *Acinetobacter* sp. SK2 when glucose + succinate are present. The expression of *hfq* in the CCR condition implicated the role of this protein in glucose and succinate utilization. Further investigations would be required to confirm participation of other regulatory genes in the regulation of MPS and other PGP traits in CCR (Hfq/sRNAs/CbrAB).

## Data Availability Statement

The raw data supporting the conclusions of this article will be made available by the authors, without undue reservation.

## Author Contributions

KB and SR conceived the idea. KB and SR designed the experimental work and performed the experiments with assistance from NG. KB and SR wrote the manuscript with support from NG. KB and NG assisted SR in critically revising the manuscript. All authors contributed to the article and approved the submitted version.

## Conflict of Interest

The authors declare that the research was conducted in the absence of any commercial or financial relationships that could be construed as a potential conflict of interest.

## Supplementary Material

Supplementary Figure 1 Screening and confirmation of *Acinetobacter* sp. SK2 mutants **(A)** Screening of mutants on LB agar with streptomycin and spectinomycin. **(B)** Selection of *gdhA*^–^ colonies on YGC medium. The *gdhA*^–^ cells lost the function of gluconic acid production, leaving no clear zone around the colonies (dashed circle). The false positive colonies still produced clear zones (solid circle). PCR confirmation. **(C)** Absence or presence of omega in wild-type and mutant strains. A band size of 2 kb consistent with the insertion of omega fragment was obtained from the mutant strains but not from the wild-type. **(D)** Verification of insertion of omega using primers designed to bind gene of interest and omega (MINF-Omega, SINF-Omega and CINF-Omega combination of primers were used to amplify omega in *gdhA*, *gdhB*, and *crc*, respectively). An increased band of approximately 2.7 to 3 kb was obtained due to the insertion of omega.

Supplementary Figure 2 Plasmid maps of recombinant vectors pKO*gdhA*, pKO*gdhB*, and pKO*crc* constructed from pKnockoutΩ. pKO*gdhA* was created by insertion of ∼500 bp *gdhA* DNA fragment (amplified from wild-type *Acinetobacter* sp. SK2) at Sa*l*I:PstI site of pKnockoutΩ. pKO*gdhB* was created by insertion of ∼540 bp *gdhB* DNA fragment at KpnI:Sa*l*I site of pKnockoutΩ. pKO*crc* contained ∼650 bp fragment of *crc* at KpnI:XbaI sites of pKnockoutΩ.

Supplementary Figure 3 Gene replacement strategy via single strand allelic exchange at the location of gene of interest (indicated as *GeneX* that can be *gdhA*, *gdhB* or *crc*). **(A)** Selection of insertional inactivated clones of GeneX (*gdhA* or *gdhB* or *crc*). The insertional inactivated mutants were screened on media containing streptomycin + spectinomycin and further confirmed by PCR. **(B)** Amplification of omega cassette from the chromosome of wild-type and mutants using primers that anneal to the omega cassette (red arrows). **(C)** Confirmation of insertion of omega cassette replacing the gene of interest. PCR amplification using primers that overlap with gene of interest (black arrow) and omega (red arrow) yield band of 2.7 to 3 kb.
