# Supplementary material for: Crc Regulates Succinate-Mediated Repression of Mineral Phosphate Solubilization in Acinetobacter sp. SK2 by Modulating Membrane Glucose Dehydrogenase
Source: Front Microbiol. 2021 Jul 12;12:641119. doi: 10.3389/fmicb.2021.641119 (PMC8312277; doi:10.3389/fmicb.2021.641119)
Supplement: Supplementary Figure 1 — Screening and confirmation of Acinetobacter sp. SK2 mutants (A) Screening of mutants on LB agar with streptomycin and spectinomycin. (B) Selection of gdhA– colonies on YGC medium. The gdhA– cells lost the function of gluconic acid production, leaving no clear zone around the colonies (dashed circle). The false positive colonies still produced clear zones (solid circle). PCR confirmation. (C) Absence or presence of omega in wild-type and mutant strains. A band size of 2 kb consistent with the insertion of omega fragment was obtained from the mutant strains but not from the wild-type. (D) Verification of insertion of omega using primers designed to bind gene of interest and omega (MINF-Omega, SINF-Omega and CINF-Omega combination of primers were used to amplify omega in gdhA, gdhB, and crc, respectively). An increased band of approximately 2.7 to 3 kb was obtained due to the insertion of omega. [file Data_Sheet_1.docx]

Supplementary Figures

**A B C D**

**M SK2 *gdhA^-^* *gdhB^-^* *crc^-^***

**M *gdhA^-^ gdhB^-^*  *crc^-^***


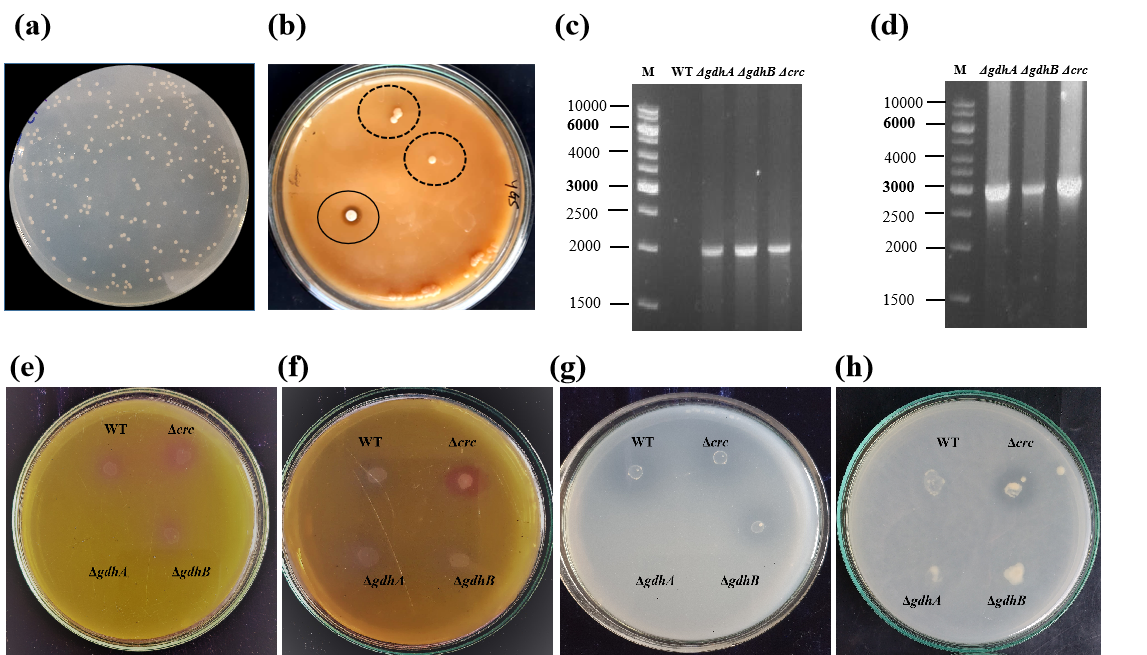


**Figure S1. Screening and confirmation of *Acinetobacter* sp. SK2 mutants (A)** Screening of mutants on LB agar with streptomycin and spectinomycin. **(B)** Selection of *gdhA^-^* colonies on YGC medium. The *gdhA^-^* cells lost the function of gluconic acid production, leaving no clear zone around the colonies (dashed circle). The false positive colonies still produced clear zones (solid circle). **PCR confirmation. (C)** Absence or presence of omega in wild-type and mutant strains. A band size of 2kb consistent with the insertion of omega fragment was obtained from the mutant strains but not from the wild-type. **(D)** Verification of insertion of omega using primers designed to bind gene of interest and omega (MINF-Omega, SINF-Omega and CINF-Omega combination of primers were used to amplify omega in *gdhA*, *gdhB* and *crc*, respectively). An increased band of approximately 2.7 to 3kb was obtained due to the insertion of omega.


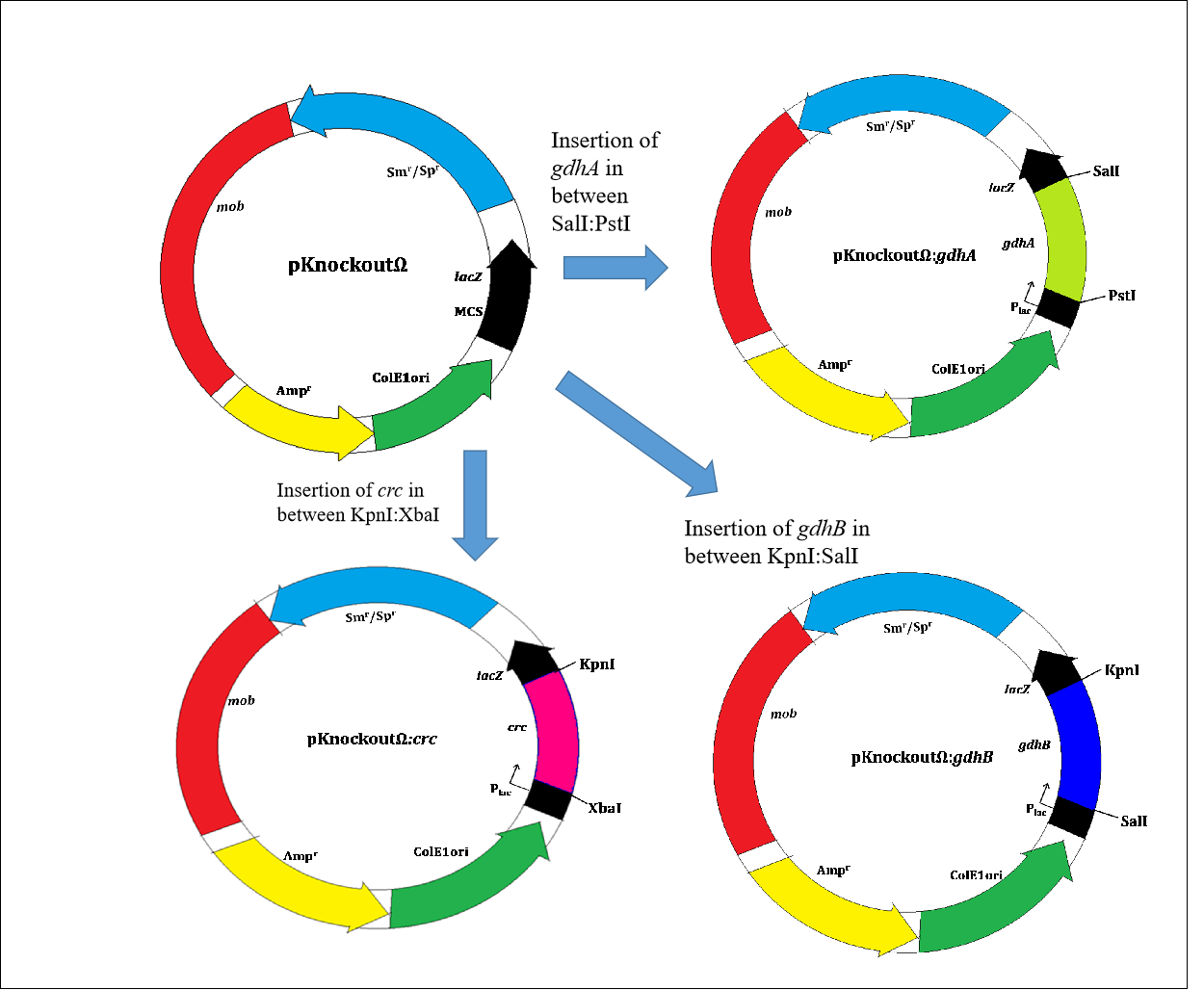


**Figure S2: Plasmid maps of recombinant vectors** pKO*gdhA*, pKO*gdhB* and pKO*crc* constructed from pKnockoutΩ*.* pKO*gdhA* was created by insertion of ~500bp *gdhA* DNA fragment (amplified from wild-type *Acinetobacter* sp. SK2) at *Sal*I:PstI site of pKnockoutΩ. pKO*gdhB* was created by insertion of ~540bp *gdhB* DNA fragment at *Kpn*I:*Sal*I site of pKnockoutΩ. pKO*crc* contained ~650bp fragment of *crc* at *Kpn*I:*Xba*I sites of pKnockoutΩ.

**C**

**B**

**A**

**M WT *gdhA^-^*  *gdhB^-^* *crc^-^***

**M *gdhA^-^*  *gdhB^-^* *crc^-^* WT**

**Figure S3: Gene replacement strategy via single strand allelic exchange at the location of gene of interest (indicated as *GeneX* that can be *gdhA*, *gdhB* or *crc*).** **(A) Selection of insertional inactivated clones of GeneX (*gdhA* or *gdhB* or *crc*)**. The insertional inactivated mutants were screened on media containing streptomycin+spectinomycin and further confirmed by PCR. (B) Amplification of omega cassette from the chromosome of wild-type and mutants using primers that anneal to the omega cassette (red arrows). (C) Confirmation of insertion of omega cassette replacing the gene of interest. PCR amplification using primers that overlap with gene of interest (black arrow) and omega (red arrow) yield band of 2.7 to 3 kb.
